# IMPACT OF CAREGIVING HOURS ON COGNITIVE OUTCOMES ACROSS ADULTHOOD

**DOI:** 10.1093/geroni/igad104.3106

**Published:** 2023-12-21

**Authors:** Kallol Kumar Bhattacharyya, Victor Molinari, Yin Liu, Elizabeth Fauth

**Affiliations:** Utah State University, Logan, Utah, United States; University of South Florida, Tampa, Florida, United States; Utah State University, Logan, Utah, United States; Utah State University, Logan, Utah, United States

## Abstract

Being a caregiver has been associated with poorer physical health and well-being. The current study expands prior work by comparing cognitive functioning among individuals who were caregivers versus non-caregivers, and further examined varying caregiving hours and cognitive functioning among caregivers. Data were from waves 2 (2003-04) and 3 (2013-14) of the Midlife in the United States (MIDUS) study. A total of 2,075 individuals aged 35-86 years (Mage=55±11) were included from wave 2. We identified individuals who served as a caregiver in the past 12 months (N=264) at wave 2. Cognitive functioning was assessed by composite scores for both executive function (inductive reasoning, category verbal fluency, working memory span, processing speed, and attention switching and inhibitory control) and episodic memory (immediate and delayed free recall of 15 words). We used multiple linear regression models to examine associations between wave 2 caregiving versus non-caregiving status on wave 3 cognitive functioning (Model 1); and, among caregivers at wave 2, varying caregiving hours and wave 3 cognitive functioning (Model 2). After controlling for covariates on sociodemographic factors, and health and functional status at baseline in Model 1, caregiving was associated with better performance in episodic memory (b=.13, p<.05), but not executive function (b=-.06, p=.09). Model 2 revealed no significant association for either episodic memory or executive function. The findings supported the healthy caregiver rather than the stress process hypothesis, and that former caregiving may be associated with better memory functioning over time among middle-aged and older adults.

